# Inappropriate asthma therapy—a tale of two countries: a parallel population-based cohort study

**DOI:** 10.1038/npjpcrm.2016.76

**Published:** 2016-10-13

**Authors:** Manon Belhassen, Anjan Nibber, Eric Van Ganse, Dermot Ryan, Carole Langlois, Francis Appiagyei, Derek Skinner, Laurent Laforest, Joan B Soriano, David Price

**Affiliations:** 1 PELyon, HESPER 7425, Claude Bernard University, Lyon, France; 2 Research in Real Life Ltd, Cambridge, UK; 3 Respiratory Medicine, Croix Rousse University Hospital, Lyon, France; 4 Centre of Population Health Studies, University of Edinburgh, Edinburgh, UK; 5 Optimum Patient Care Ltd, Cambridge, UK; 6 Instituto de Investigación Hospital Universitario de la Princesa (IISP), Universidad Autónoma de Madrid, Cátedra UAM-Linde, Madrid, Spain; 7 Centre of Academic Primary Care, University of Aberdeen, Aberdeen, UK

## Abstract

Against recurrent controversies around the safety of short- and long-acting β_2_-agonists (SABA and LABA), and the National Review of Asthma Deaths inquiry in the United Kingdom, we investigated the prevalence of inappropriate therapy in asthma. Our study aimed to determine the prevalence of inappropriate use of asthma therapy in the United Kingdom and in France. Two interval, parallel, population-based cohorts (2007 and 2013) were developed in each country by using the UK OPCRD and the French EGB databases. Patients aged 6–40 years were studied over the 12-month period following inclusion, regarding overuse (⩾12 units) of SABA, use of LABA without inhaled corticosteroids (ICS) and ⩾2-fold higher use of LABA compared with that of ICS. Overall, 39,743 UK and 4,910 French patients were included in 2007, and 14,036 and 5,657 patients, respectively, were included in 2013. UK adults were more frequently exposed to SABA overuse compared with those in France in both periods, with an upward trend in the United Kingdom (*P*<0.05). In 2013, LABA use without ICS occurred in 0.1% and 1.5% of United Kingdom and French adults, respectively. Unbalanced use of LABA relative to ICS became marginal in both countries in 2013. Inappropriate use of therapy was less marked, but present, in children. Inappropriate therapy remains a common issue in asthma. Based on our figures, it may be estimated that >210,000 British and >190,000 French asthmatics aged 6–40 years were inappropriately treated in 2013.

## Introduction

Potentially preventable factors have been identified in the majority of asthma exacerbations and deaths.^1^ In addition, despite widely disseminated guidance for asthma management,^2^ intensive research concerning asthma management in primary care, novel drugs for treatment,^3^ and research on effectiveness,^4^ morbidity and mortality due to asthma (in all age categories) remains unacceptably high throughout Europe and elsewhere, with the recently updated figure of 334 million asthmatics worldwide in 2012.^5^

Contributing factors include the inappropriate prescribing and use of asthma medication. A recent report from the United Kingdom, prepared by the National Review of Asthma Deaths, aimed to identify avoidable risk factors and to make recommendations for improving care and reducing the number of deaths.^6^ This report drew attention to the excessive prescribing of reliever medication (39% of patients had been prescribed >12 short-acting reliever inhalers in the year before they died), or the under-prescribing of preventer medication; namely, 38% of patients were issued with fewer than 4 and 80% with fewer than 12 preventer inhalers in the previous year, and the inappropriate prescribing of long-acting β_2_-agonist (LABA) bronchodilator inhalers (14% of those who died were prescribed a single-component LABA bronchodilator at the time of death and at least 3% of patients were on LABA monotherapy without inhaled corticosteroid (ICS) preventer treatment). Against this background, we investigated asthma therapy in comparable populations of the United Kingdom and France, to assess the prevalence of inappropriate use and to identify changes over recent years (2007–2013).

## Results

In 2007, 39,743 UK patients were included in the cohort, with a mean age of 23.7 years (27% children and 73% adults) and 53.2% males. In 2013, 14,036 patients met our inclusion criteria, with a mean age of 24.2 years (22% children and 78% adults) with 51.8% males.

In 2007, 4,910 French patients were studied. The cohort included 50.8% males (33% children and 66% adults), with a mean age of 22.1 years. In 2013, 5,657 patients were included (35.5% children and 64.5% adults), with a mean age of 21.4 years with 51.1% males (Table 1).

### Inappropriate patterns of use

#### SABA overuse

**Figure 1:**
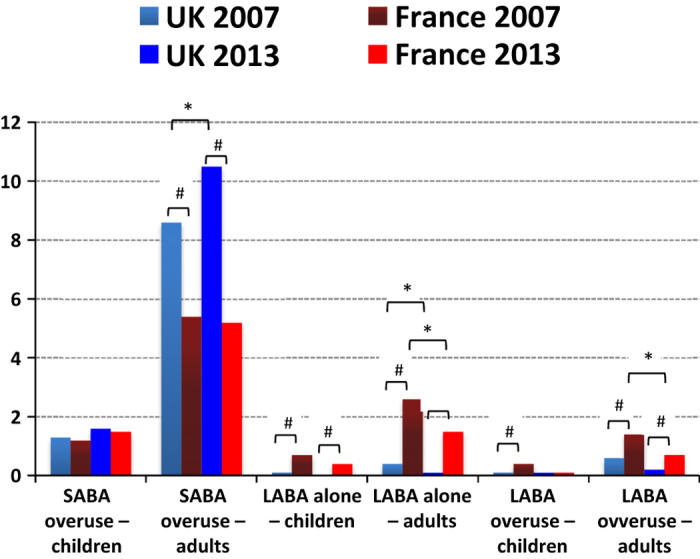
Frequency (%) of inappropriate asthma therapy use in UK and French patients during the 12-month follow-up. *P value<0.05 when comparing 2007 versus 2013. ^#^ *P* value<0.05 when comparing UK versus France.

Short-acting β_2_-agonist (SABA) overuse in 2013, defined as 12 prescriptions/dispensations or more over 12 months, was identified in 1.6% and 1.5% of the UK and French children, respectively. These percentages were slightly increased compared with those in 2007, but the difference was not statistically significant (1.3% and 1.2%, respectively*; P*=0.37 for France and *P*=0.19 for the United Kingdom). UK adults had higher levels of inappropriate SABA use, both in 2007 and in 2013, than French patients (*P*<0.0001 for both years). In 2007 and 2013, 8.6% and 10.5% of adult UK patients were exposed to inappropriate SABA use, respectively. This was the case for 5.4% and 5.2% of French patients in 2007 and 2013, respectively (Figure 1). Overall, SABA overuse in adults had increased in the United Kingdom (*P*<0.001), although remaining stable in France (*P*=0.67).

#### LABA inappropriate use

In 2013, inappropriate LABA use occurred in 0.1% and 1.5% of UK and French adults, respectively. This was a large reduction from 2007 UK and French figures of 0.4% and 2.6%, respectively (*P*<0.05 for both). Excessive use of LABA relative to ICS (except fixed-dose combination (FDC)) occurred in 0.2% of UK adults and in 0.7% of French adults in 2013. These percentages decreased from the 2007 figures of 0.6% and 1.4% for United Kingdom (*P*=0.29) and France (*P*=0.003), respectively (Figure 1), but the difference was statistically significant for the French figures only. Results in children showed downward trends by time in both countries, although compared with SABA overuse, LABA inappropriate use was more frequent in French than in UK asthmatic children (*P*<0.0001).

### Extrapolation to the whole UK and French populations

Altogether, in 2013, in the age group 6–40 years, the number of UK and French patients at risk because of inappropriate asthma management could be estimated at ~210,000 and 190,000, respectively, overwhelmingly due to SABA overuse. In the United Kingdom, SABA overuse increased between 2007 and 2013, whereas it remained stable in France. On the contrary, inappropriate use of LABA, both in France and in the United Kingdom, declined considerably between 2007 and 2013 (Table 2; Figure 1).

## Discussion

### Main findings

In this study analysing primary care data in 2007 and 2013, we identified, both in the United Kingdom and France, numerous patients inappropriately treated for their asthma, and therefore at risk of adverse outcomes. Altogether, based on our figures and on known asthma prevalence, it was estimated that in 2013 at least 210,000 and 190,000 patients were exposed to inappropriate patterns of asthma therapy in the United Kingdom and France, respectively, with a major concern regarding SABA overuse. Indeed, in the United Kingdom, SABA overuse increased between 2007 and 2013 (12.1% of asthma patients), whereas it remained stable in France (3.9%). Children were also overexposed to SABA, with 1.2% in the United Kingdom and 1.5% in France with 12 or more SABA units prescribed/dispensed per year.

Based on our study, extrapolating to the national population suffering from asthma (within the 6–40-year age band only), in 2013 in the United Kingdom at least 7,000 asthma patients used LABA inadequately. This was LABA use either with no ICS co-therapy at all, or with periods of use of LABA as monotherapy, a risk factor for severe asthma attacks and death.^7^ In France, the situation was even more worrying, with 55,000 asthma patients inappropriately exposed to LABA in 2013. Nonetheless, large improvements were observed between 2007 and 2013 regarding LABA use in both countries.

### Interpretation of findings in relation to previously published work

Many studies have reported that regular use of SABA is associated with increased airway hyper-responsiveness,^8^ mortality,^9^ healthcare utilisation^10^ and poor asthma control.^11^ Consequently, current asthma treatment guidelines^2^ recommend using quick-relief medications on an as-needed basis rather than as a regularly scheduled medication. Despite these recommendations and well-documented adverse consequences of excessive SABA use, our data are in line with other studies suggesting that overuse of SABA, misuse of LABA and underuse of ICS are common, and they may compromise patient health.^10,12^

The safety of LABA remains controversial in asthma, particularly in children, which led regulators to contraindicate LABA as a single agent in asthma treatment from 2010.^13^ Recently updated meta-analyses for formoterol and salmeterol failed to conclude concerns on safety.^7,14^ Despite the absence of evidence of serious risk with LABA associated with ICS in RCTs, likely due to infrequent occurrence of many events, the precision of results was low. Evidence is also limited in the observational context. Consequently, the Food and Drug Administration issued in 2010 a ‘Dear Doctor Letter’ to warn of the risks associated with prescribing LABA alone for asthma.^15^ The improvements in LABA use observed in both countries may be the consequence of such regulatory warnings. Part of LABA use in monotherapy could also be due to the prevention of exercise-induced asthma.^16^ However, as such instances are rare, the overwhelming majority of LABA users should use ICS simultaneously (i.e., in fixed-dose combinations).

### Strengths and limitations of this study

This study is important as it explores inappropriate patterns of asthma therapy use in longitudinal data sets comprising large numbers of patients. These data sets exhaustively record all therapies prescribed or dispensed. A limitation in the use of claims data was, however, the absence of disease diagnostic codes in France, so that asthma patients were identified using an algorithm (age criteria and dispensations). However, based on this algorithm, our asthma prevalence data were comparable to the data from other studies.^17,18^ By contrast, in the United Kingdom, asthma patients were diagnosed using a physician-diagnostic code.^19,20^ Quality of asthma diagnosis may however be debatable in the primary care setting in the absence of referral to a specialist, and over-diagnosis and misdiagnosis of asthma are a possibility.^2,21^

In our study, additional precautions were taken to include patients likely to suffer from asthma, including the requirement of at least three dispensations of R03 over 12 months in a prior period. In addition, children aged <6 years, adults >40 years (to avoid including chronic obstructive pulmonary disease (COPD)) and patients with cystic fibrosis were excluded. Consequently, our study was completed with cohorts that were highly likely to include only asthma patients.

Two different data sets were used, one recording prescribing and the other recording dispensations. Unfilled prescriptions are not recorded in French claims data, and there was no way to verify whether inadequate use was primarily due to patients or to healthcare professionals. However, one of our recent studies has shown that inappropriate management by general practitioners is not uncommon in France or in the UK.^22^

No outcome data (e.g., deaths and hospitalisations) were computed, as the purpose of this research was to give an overview of the extent of inappropriate management of asthma. Outcome data will be collected in the context of the ASTROLAB study that relates detailed patterns of use of asthma therapy to outcomes.^23^

Another issue raised by the UK National Review of Asthma Deaths was the underuse of ICS. In this study, underuse of ICS was not investigated, as unlike overuse of SABA and use of LABA without ICS, this exposure is difficult to assess in isolation from the clinical context. Beyond the complex issue of adherence,^24^ many subjects have seasonal symptoms leading to temporary ICS use. In the absence of detailed clinical data and within our observational study design, it is almost impossible to distinguish persistent asthma, in need of regular ICS therapy, from milder or intermittent asthma, at lower risk of exacerbations.

### Implications for future research, policy and practice

The prevalence of inappropriate patterns of use of asthma therapy was high, particularly regarding the overuse of SABA. The estimate was higher in the United Kingdom, possibly due to the insufficient priority given to respiratory diseases in the United Kingdom.^25^ Inappropriate LABA use confers considerable risk, both for patients exposed to LABA only, and for patients with at least twice higher use of LABA than of single ICS.

Clinicians could improve the quality of prescribing,^22^ and patients should be trained regarding appropriate use of therapy^24^ and technical use of inhalers.^26^ Clinicians should be informed of the actual exposure of their patients, e.g., via feedback from claims data or from consulting practice prescribing records. Personal action plans should be written, describing how patients may recognise a deterioration in their asthma and what steps they should take to re-establish control.^2^ In the United Kingdom, SIMPLES is a structured primary care approach to the structured review of patients with partly controlled or uncontrolled asthma, which encompasses patient education monitoring, lifestyle and pharmacological management while addressing support needs.^27^ The SIMPLES approach, used iteratively, should be able to identify those patients who might benefit from referral to a specialist for further assessment avoiding inappropriate escalation of treatment. Other clinical healthcare professionals (e.g., nurses) could also review the use of therapy, and provide training and support to patients.^28^

Policymakers should develop actions targeting both clinicians (asthma audits) and patients (e.g., providing ways for patients to have direct contacts with advisors, e.g., using IT, or through pharmacies). In the United Kingdom, from their electronic medical records, general practitioners can easily access the list of prescribed asthma treatments, enabling identification of those who should be invited to attend for regular reviews. A similar approach could improve the quality of asthma management in France.

### Conclusions

Concomitant outcome data would be useful to illustrate the potential impact of inappropriate management in asthma, and to assess the quality of care in large asthma populations. Other markers of quality of care and predictors of subsequent asthma exacerbation should not be overlooked (e.g., low therapeutic ratio controller-to-total asthma drug ratio or ICS-to-total asthma drug ratio (<50%)).^29^

This study might be replicated targeting populations at high risk, such as patients who experienced near-fatal asthma, or more generally those who have experienced asthma-related hospitalisation, or used several courses of oral corticosteroids over 1 year.

Using similar methods, the study might be conducted in other European countries, to identify inappropriate patterns of use and to improve the care of asthma.

Holistic interventions on general practitioners and on patients’ behaviours might be also conducted.^30^ Systematic targeting of education and skills training in primary care have demonstrated the ability to make significant impact on patient outcomes.^31^ Finally, omics and other system approaches within existing projects^32^ might be considered.

We conclude that both in the United Kingdom and France, despite noticeable differences in healthcare systems, inappropriate asthma therapy is a frequent finding that improved little during the past decade. Therefore, concerns are raised about the current delivery of asthma care, as individual patients are exposed to potentially serious, but preventable, outcomes. Corrective measures including educational interventions, and close monitoring at the population level, are deemed necessary to reduce the asthma-associated burden.

## Materials and Methods

### Setting

Parallel population-based cohorts were created in each country for two distinct periods: 2007 and 2013. The Optimum Patient Care Research Database (OPCRD) electronic health records database in the United Kingdom and the Permanent Beneficiaries Sample database (EGB) in France were used.

The OPCRD comprises routinely recorded clinical data extracted through the Optimum Patient Care Clinical Service Evaluation. The service extracts data from 600 practices spread throughout the United Kingdom, and collects all longitudinal data from these practices. As new practices are being recruited and contribute data from past years, this database resulted in a higher data collection from 2007 versus 2013. The database (anonymised) is comprised of electronic medical records and characterises patients in terms of their demography, disease control and exacerbation history.^33^

The EGB database is a 1/97th representative random sample of the *Système National d'Information Inter-Régimes de l'Assurance Maladie* (SNIIR-AM). The SNIIR-AM^34^ is a French nation-wide, population-based database that records anonymised individual data on all reimbursements for healthcare utilisation, including therapy. There is no information on the medical condition linked to reimbursements.

### Selection criteria

Patients with asthma were selected in both countries, for the years 2007 and 2013. Given the differences between the database sources used, notably regarding the availability of recorded diagnoses, specific inclusion criteria were used for each country, to allow comparison. In the United Kingdom, patients with a diagnosis of asthma in 2007, plus at least three prescriptions of respiratory drugs (SABAs, LABAs, ICS, fixed-dose combination of ICS and LABAs, leukotriene receptor antagonist and xanthines) at three different dates (entry date=date of third prescription), and aged 6–40 years at entry date were selected.

French patients with at least three dispensations of respiratory drugs (SABAs, LABAs, ICS, fixed-dose combination of ICS and LABAs, leukotriene receptor antagonist and xanthines) in 2007 at three different dates were qualified to enter the cohort (entry date=date of the third dispensation). Patients aged 6–40 years at entry date were selected. An upper age limit of 40 was selected to avoid including patients with COPD or other age-related respiratory conditions.

The same exclusion criteria were used for both countries during the 12 months before entry date, namely: any dispensation/prescription of omalizumab, chronic use of oral corticosteroids (defined as >4 prescriptions of oral steroids at ⩾4 different dates over 12 months preceding entry date in the UK OPCRD, and as ⩾4 different dates of dispensation of oral steroids over 12 months at ⩾2 different quarters in French EGB), the presence of a diagnosis of COPD or prescribing/dispensation of tiotropium or indacaterol alone, cystic fibrosis, lung cancer, bronchiectasis, tuberculosis or sarcoidosis. Patients with <12 months of follow-up as of entry date were also discarded. In France, exclusion criteria were obtained from long-term condition (LTC) status and/or hospital admission (diagnoses in ICD-10 codes) and on Anatomical Therapeutic Chemical codes. In the United Kingdom, exclusion criteria were assessed from Read codes based on physician diagnoses^35^ (Appendix available upon request to the authors).

### End points

The occurrence of three inappropriate patterns of use of asthma therapy was assessed during the study period:

1. Overuse of SABAs, defined as 12 units or more over 12 months,
  - Either LABA without ICS (single ICS or in fixed-dose combination LABA+ICS).
  - Or unbalanced use of LABA with respect to ICS use, defined as at least twice as many units of LABA prescribed/dispensed than units of single ICS (e.g., ratio LABA/ICS⩾2).
2. Inappropriate (off-label) LABA use, defined as:
  - Either LABA without ICS (single ICS or in fixed-dose combination LABA+ICS).
  - Or unbalanced use of LABA with respect to ICS use, defined as at least twice as many units of LABA prescribed/dispensed than units of single ICS (e.g., ratio LABA/ICS⩾2).

Treatment units consisted of prescribed/dispensed packs.

### Statistical analysis

Parallel analyses were conducted for both 2007 and 2013 cohorts. Analyses were stratified by age groups: children (6–13 years) and adults (14–40 years).

Patients were preliminarily described according to baseline characteristics at cohort entry.

In each country, patients’ characteristics and inappropriate patterns were described in 2007 and 2013 cohorts. The British and French 2013 cohorts were compared as to the percentages of patients presenting each criterion during the study period.

Finally, an extrapolation of asthma burden was estimated in the general population via direct standardisation.^36^ For the United Kingdom, extrapolations were computed by applying the prevalence of asthma (computed from Quality Outcome Framework recorded prevalence) in the entire UK population for the given year to the number of UK subjects within the 6–40-year age band (taken from the Office for National Statistics).^37^ Extrapolations to the overall French asthma population aged 6–40 years were computed based on the representativeness of the EGB, i.e., random sample of the French population^38^ and the prevalence of asthma in patients aged 6–40 years.^17^

Quantitative variables were described using descriptive statistics: means and s.d.’s, and qualitative variables with counts and percentages. Comparative statistics were conducted with Mann–Whitney tests for continuous variables, and *Χ* ^2^-tests for percentages. Statistical significance was considered at *P* value<0.05.

## Figures and Tables

**Table 1:** Patients baseline characteristics

	*2007*		*2013*	
	*UK* * (*N*=39,743)*	*France (*N*=4,910)*	*UK* * (*N*=14,036)*	*France (*N*=5,657)*
Mean age	23.7 (11.09)	22.1 (11.40)	24.2 (10.69)	21.4 (11.14)
*Age groups (*N*, %)*				
Children (6–13 years)	10,776 (27.1%)	1,655 (33.7%)	3,138 (22.4%)	2,009 (35.5%)
Adults (14–40 years)	28,967 (72.9%)	3,255 (66.3%)	10,898 (77.6%)	3,648 (64.5%)
Males (*N*, %)	21,142 (53.2%)	2,494 (50.8%)	7,272 (51.8%)	2,916 (51.5%)

**Table 2:** Extrapolation of the number of subjects with inappropriate asthma therapy use to the UK and French population (aged 6–40 years)

*Extrapolations*	*2007*		*2013*	
	*UK*	*France*	*UK*	*France*
SABA overuse	158,560	139,650	206,040	136,150
LABA without ICS	7,040	68,600	2,720	38,500
LABA relative overuse with respect to ICS use	11,520	38,500	4,760	17,150
Total	177,120	246,750	213,520	191,800

Abbreviations: ICS, inhaled corticosteroid; LABA, long-acting β_2_-agonist; SABA, short-acting β_2_-agonist.
